# Plant Responses to Climate Change

**DOI:** 10.3390/ijms242115902

**Published:** 2023-11-02

**Authors:** Isabel Marques, José C. Ramalho, Ana I. Ribeiro-Barros

**Affiliations:** 1 Plant-Environment Interactions and Biodiversity Lab, Forest Research Centre & Associate Laboratory TERRA, Higher School of Agriculture, University of Lisbon, 1349-017 Lisboa, Portugal; cochichor@isa.ulisboa.pt (J.C.R.); anaifribeiro@campus.ul.pt (A.I.R.-B.); 2 GeoBioSciences, GeoTechnologies and GeoEngineering, Faculdade de Ciências e Tecnologia, Universidade NOVA de Lisboa, 2829-516 Monte de Caparica, Portugal

## 1. Molecular Mechanisms of Plants to Climate Change

Ongoing climate change poses a great risk to the natural environment and the sustainability of agriculture [1,2]. Major abiotic stresses, such as extreme temperatures and drought, are already responsible for 51% to 82% of global annual losses in crop yield, a scenario expected to be aggravated in the future [3,4,5]. Thus, scientific advances have a special role in meeting the challenges of overcoming such impacts. In this context, this Special Issue covers basic and applied research aimed at understanding the molecular mechanisms associated with plant responses to abiotic stresses, including drought, cold, heat, high light, and salinity. A particular emphasis was placed on the influence of stresses at the whole genome or the transcriptome level, the characterization of tolerance across different accessions and genotypes, and the discovery of candidate genes that can improve the productivity and yield of crops under changing abiotic conditions. The role of soil microbiome in enhancing plant tolerance against stresses has also been revised. Taken together, the new information provided in these manuscripts not only increases our understanding of the molecular basis of plants’ adaptive responses but also provides key fundamentals for the future successful selection and breeding of tolerant crops.

## 2. List of Contributions

Twenty-four manuscripts were submitted for consideration for this Special Issue, and after peer review, ten of them were finally accepted for publication (nine articles and one review). These studies were performed in multiple scientific institutions from Brazil, Croatia, China, Egypt, Portugal, Russia, the USA, and Vietnam. The contributions are listed below:

1. Zhao, W.; Song, J.; Wang, M.; Chen, X.; Du, B.; An, Y.; Zhang, L.; Wang, D.; Guo, C. Alfalfa MsATG13 Confers Cold Stress Tolerance to Plants by Promoting Autophagy. Int. J. Mol. Sci. 2023, 24, 12033. https://doi.org/10.3390/ijms241512033.
2. Lu, Z.; Liu, H.; Kong, Y.; Wen, L.; Zhao, Y.; Zhou, C.; Han, L. Late Elongated Hypocotyl Positively Regulates Salt Stress Tolerance in *Medicago truncatula*. Int. J. Mol. Sci. 2023, 24, 9948. https://doi.org/10.3390/ijms24129948.
3. Marques, I.; Fernandes, I.; Paulo, O.S.; Batista, D.; Lidon, F.C.; Partelli, F.; DaMatta, F.M.; Ribeiro-Barros, A.I.; Ramalho, J.C. Overexpression of Water-Responsive Genes Promoted by Elevated CO_2_ Reduces ROS and Enhances Drought Tolerance in *Coffea* Species. Int. J. Mol. Sci. 2023, 24, 3210. https://doi.org/10.3390/ijms24043210.
4. Bauer, N.; Tkalec, M.; Major, N.; Talanga Vasari, A.; Tokić, M.; Vitko, S.; Ban, D.; Ban, S.G.; Salopek-Sondi, B. Mechanisms of Kale (*Brassica oleracea* var. *acephala*) Tolerance to Individual and Combined Stresses of Drought and Elevated Temperature. Int. J. Mol. Sci. 2022, 23, 11494. https://doi.org/10.3390/ijms231911494.
5. Yuan, P.; Poovaiah, B.W. Interplay between Ca2+/Calmodulin-Mediated Signaling and *AtSR1*/*CAMTA3* during Increased Temperature Resulting in Compromised Immune Response in Plants. Int. J. Mol. Sci. 2022, 23, 2175. https://doi.org/10.3390/ijms23042175.
6. Islam, M.M.; Qi, S.; Zhang, S.; Amin, B.; Yadav, V.; El-Sappah, A.H.; Zhang, F.; Liang, Y. Genome-Wide Identification and Functions against Tomato Spotted Wilt Tospovirus of PR-10 in *Solanum lycopersicum*. Int. J. Mol. Sci. 2022, 23, 1502. https://doi.org/10.3390/ijms23031502.
7. Wan, H.; Qian, J.; Zhang, H.; Lu, H.; Li, O.; Li, R.; Yu, Y.; Wen, J.; Zhao, L.; Yi, B.; et al. Combined Transcriptomics and Metabolomics Analysis Reveals the Molecular Mechanism of Salt Tolerance of Huayouza 62, an Elite Cultivar in Rapeseed (*Brassica napus* L.). Int. J. Mol. Sci. 2022, 23, 1279. https://doi.org/10.3390/ijms23031279.
8. Luo, Y.; Teng, S.; Yin, H.; Zhang, S.; Tuo, X.; Tran, L.-S.P. Transcriptome Analysis Reveals Roles of Anthocyanin- and Jasmonic Acid-Biosynthetic Pathways in Rapeseed in Response to High Light Stress. Int. J. Mol. Sci. 2021, 22, 13027. https://doi.org/10.3390/ijms222313027.
9. Bazhenov, M.S.; Chernook, A.G.; Bespalova, L.A.; Gritsay, T.I.; Polevikova, N.A.; Karlov, G.I.; Nazarova, L.A.; Divashuk, M.G. Alleles of the GRF3-2A Gene in Wheat and Their Agronomic Value. Int. J. Mol. Sci. 2021, 22, 12376. https://doi.org/10.3390/ijms222212376.
10. Chen, Y.; Yao, Z.; Sun, Y.; Wang, E.; Tian, C.; Sun, Y.; Liu, J.; Sun, C.; Tian, L. Current Studies of the Effects of Drought Stress on Root Exudates and Rhizosphere Microbiomes of Crop Plant Species. Int. J. Mol. Sci. 2022, 23, 2374. https://doi.org/10.3390/ijms23042374.

Contribution 1 explored the role that autophagy-related genes (ATGs) have in regulating and even improving plant tolerance to abiotic stresses. Autophagy is an evolutionarily conserved self-degradative process in all eukaryotic cells and plays many physiological roles in maintaining cellular homeostasis. However, the biological mechanisms and action of *ATGs* under abiotic stresses remain unclear. Specifically, the authors showed how the up-regulation of the gene *MsATG13* enhanced cold tolerance in alfalfa (*Medicago sativa* L.) through the modulation of autophagy and antioxidant levels. This gene seems to activate other key *ATGs*, increasing antioxidant levels and reducing the accumulation of ROS in response to cold. These results demonstrate that autophagy plays an important role in plant survival under abiotic stress.

Contribution 2 revealed how the core component of the circadian clock, *MtLHY* (the Late Elongated Hypocotyl orthologue) is responsible for salt stress tolerance in *Medicago truncatula*. The circadian clock is an internal time mechanism that synchronizes the physiological response of an organism to its surroundings, including changing environmental conditions. In this study, the authors showed how the expression of *MtLHY* increases salt stress tolerance in *M. truncatula* through flavonoid biosynthesis, while mediating Na^+^ /K^+^ homeostasis and inhibiting ROS production. These results show an existing link between the circadian clock and plant responses to stresses.

Contribution 3 studied the single and combined effects of drought and elevated air [CO_2_] (eCO_2_) on the transcriptomic machinery of two *Coffea* genotypes (from the two species that support global coffee trade): *Coffea canephora* Pierre ex *A. Froehner* cv. Conilon Clone 153 (CL153) and *C. arabica* L. cv. Icatu Vermelho (Icatu). The authors showed a predominance of protective and ROS-scavenging genes, directly or indirectly related to ABA signaling pathways involved in tolerance responses, although with different regulatory mechanisms in Icatu and CL153. Moderate drought had a minor impact on the number of transcripts differentially regulated in these plants, contrary to severe water deficit. Additionally, it was found that elevated eCO_2_ attenuated the impacts of drought in the two genotypes but especially in Icatu, in agreement with the contrasting physiological tolerance responses previously reported in these genotypes.

Contribution 4 explored the effects of individual and combined stress (osmotic + heat stress) in the tolerance responses of 33 kale accessions (*Brassica oleracea* var. *acephala*). The authors showed that the more tolerant accessions had higher basal contents of proline, total soluble sugars, glucosinolates, and heat shock proteins and higher transcript levels of *NAC* and *DREB* transcription factors. On the contrary, sensitive accessions were characterized by a high basal content of fructans. These findings can be further used as markers for screening heat- and drought-tolerant kale cultivars.

Contribution 5 reported the effects that a moderate change in temperature had on plant immune response, through Ca^2+^/calmodulin-mediated signaling. Plants use hormones to respond to environmental changes. Ca^2+^ signaling plays an important role in the adaptation to abiotic stresses, but the underlying mechanisms involved in this process are still scarcely known. The authors showed how *AtSR1*/*CAMTA3*, a Ca^2+^/CaM receptor, is involved in increased temperature-mediated stomatal defense and apoplastic immunity. Together with other studies, this suggests that Ca^2+^ signaling acts as a general defense response to pathogen infection in the context of temperature.

Contribution 6 was focused on the action of pathogenesis-related (PR) proteins under pathogen attack or infection. Specifically, the authors aimed to understand if these genes act to defend tomato plants against infection by the spotted wilt virus (TSWV), one of the most important plant viruses in the world. This study identified and characterized forty-five candidate genes, with some, such as *PR-10* and *Sw-5b*, up-regulated upon infection. These findings lead to a better understanding of how systemic necrosis occurs upon infection by the tomato spotted wilt tospovirus, which could aid in the future development of new antiviral approaches.

Contribution 7 explored the molecular mechanism of salt tolerance in rapeseed (*Brassica napus* L.), comparing two varieties with significant differences in salt tolerance: Zhongshuang 11 (ZS11), a conventional rapeseed variety, which is sensitive to salt stress, and Huayouza 62 (H62), an elite rapeseed cultivar, widely planted in China. Several differentially expressed metabolites and key differentially expressed genes were identified through metabolomics and transcriptomics analysis, revealing vast differences in the two varieties and a high mechanism of tolerance to salt, especially in H62. Specifically, several candidate genes of non-specific lipid transporters (nsLTPs) were identified as important for salt tolerance since they were significantly up-regulated as the salt concentration increased, especially in the salt-tolerant H62.

Contribution 8 studied the molecular mechanisms underlying rapeseed’s response to high light (HL) stress, namely the potential roles of anthocyanins and jasmonic acid (JA) in plant adaptations. Although light is an essential environmental factor for plant growth and development, HL is also a stress factor, although scarcely studied. This work showed that plants under HL, up-regulated genes that were involved in the regulation and biosynthesis of anthocyanins and JA. In accordance, the accumulation of anthocyanins, which act as photoprotectants, was significantly promoted under HL conditions. In agreement with the positive regulatory role of JA in anthocyanin biosynthesis, this study suggests that JA plays a key role in the responses of rapeseed seedlings to HL, contributing to the development of HL-tolerant rapeseed plants.

Contribution 9 studied how the allelic diversity of growth-regulating factors (GRF), a family of plant-specific transcription factors with roles in plant growth, development, and stress response, changed between bread wheat (*Triticum aestivum* L.) and ancestral wheat accessions. The authors identified a rare allele in a world wheat collection, associated with earlier heading and higher grain filling, which is suggested to be an ancestral adaptation of local landraces in the Black Sea region. Together with the finding of other unique mutations, this study reveals the importance of ex situ conservation for the preservation of unique genetic alleles.

Contribution 10 reviewed the impact of drought stress on root exudates and associated microbiomes, discussing how they help plants to successfully respond to drought. The authors highlight how root exudates play an important role in the recruitment of mycorrhizal fungi and plant growth-promoting rhizobacteria (PGPR), with implications for plant tolerance against environmental stresses. However, the crosstalk between root-associated microbiomes and root exudates, as well as the construction of beneficial microbial consortia in agriculture still presents great challenges. Therefore, a deeper understanding of root exudates and soil microbiomes is vital for disentangling their role in plant fitness and sustainable agriculture.

## 3. Future Perspectives

This Special Issue progresses our understanding of the molecular mechanisms underlying the responses of plants to abiotic stresses and highlights that multiple and complex processes are involved, including signaling pathways, transcription, translation, and post-translational modifications. Despite the remarkable research involved in this Special Issue combining genomics, transcriptomics, genetics, and other approaches, there are still much scope for plant research. Further studies should clarify the key relationships between abiotic stresses and how they interact individually and in combination [3]. Current research on plant tolerance is also, generally, focused on vegetative parts. Therefore, more studies are needed to understand the impacts of abiotic stressors on other key plant components such as the flowers, which are highly sensitive to environmental changes [6]. Together with emerging solutions such as genome editing and the advance in artificial intelligence, this would be key in aiding sustainable agriculture and the food requirements of the ever-increasing human population.
